# Promoting physical activity among community-dwelling seniors living in a Francophone rural area in New Brunswick: a pre-implementation qualitative study

**DOI:** 10.3389/fpubh.2025.1498397

**Published:** 2025-04-09

**Authors:** Jalila Jbilou, Sharmeen Jalal Chowdhry, Joey Frenette, Iza Pinette Drapeau, Ellène Comeau, Adrien Bouhtiauy, Saïd Mekari

**Affiliations:** ^1^School of Psychology, Université de Moncton, Moncton, NB, Canada; ^2^Centre de Formation Médicale du Nouveau-Brunswick, Université de Sherbrooke-Moncton Campus, Sherbrooke, QC, Canada; ^3^Department of Medicine, Université de Sherbrooke, Sherbrooke, QC, Canada

**Keywords:** community-dwelling seniors, physical activity, rural, qualitative design, Canada

## Abstract

**Objectives:**

This study aimed to gather an in-depth understanding of Francophone community-dwelling seniors’ needs and expectations regarding physical activity to inform the design and implementation of a community-based program in a rural area in New Brunswick.

**Methods:**

Using the socioecological model, a qualitative design was co-created and an interview guide co-developed to collect data from 24 participants, including two focus groups and 13 individual interviews. Content analyses were carried out to categorize and conceptualize the data into main and subthemes.

**Results:**

Four major themes emerged, including the presence of challenges and barriers (community and environmental obstacles, personal challenges, and social or cultural challenges), motivators and incentives (demographics, understanding the benefits of the program, sense of belonging, and preferred physical activities), designing program infrastructure (How, What, Where, time of offer, evaluation of capabilities, feelings of familiarity), and strategies to improve recruitment and retention (what would best allow participants to join and remain in the program). The findings of this study highlighted the key challenges community-dwelling seniors living in a rural area face in participating in physical activity programs (i.e., personal issues, geographic aspects, the importance of physical capacities, and cultural trends).

**Conclusion:**

While codesigning physical activity programs for community-dwelling seniors living in rural areas is time-consuming, it allows for a better understanding of the social and organizational assets and challenges of the target community. It also strategically contributes to managers’ ownership and community engagement of/for the program to support its implementation and promotion.

## Introduction

The aging population in Canada is growing rapidly. In 2023, 18.5% of the Canadian population was aged 65 years and over (1). By 2030, the proportion of this population is projected to increase to between 21.3 and 22.9% (2). In New Brunswick (NB), an officially bilingual and small province, the population aged 65 and over comprised 23% of the total population in 2023 (3). Notably, Statistics Canada projects that NB will have one of the highest proportions of older adults (those aged 85 and over) in the country, particularly within the Atlantic provinces (2). In addition, about 40.7% of NB seniors live in rural and remote communities and 19.5% are Francophones (3).

Promoting healthy aging and enhancing the quality of life for seniors are paramount concerns, and physical activity (PA) plays a central role in achieving these objectives. Rural areas in Canada face significant challenges regarding access to preventative community-based programs. Access to supportive settings for PA, an essential means of incorporating PA into one’s daily routine, is a significant issue. A lack of policy on PA associated with the physical, natural, and built environments in rural communities is a significant threat to health equity in Canada, especially for older people (4). In addition, socioeconomic factors also play an important role in the PA and the built environment among adults in Canada (5).

In 2020, one in two New Brunswickers aged 65 and over participated in moderate or vigorous physical activities at least two and a half hours per week (6). In NB, Canada, promoting PA among community-dwelling seniors is particularly vital due to its potential to mitigate the adverse effects of sedentary behavior and improve overall health outcomes. Several studies relate the PA for this population to the physical or built environment and community neighborhood characteristics in an urban setting (7), such as the importance of parks (8), open green spaces (9), and walkability (10).

Francophone seniors are at heightened risk for adverse health outcomes. Francophone seniors tend to have worse perceptions of their health than seniors of other languages (11). This notion holds some truth when considering that this population is more frequently confronted with chronic diseases (i.e., asthma, high blood pressure) (12). In addition, Francophone seniors face diverse challenges when attempting to better their health, such as not being able to speak their language with their doctors (11, 12). Moreover, they tend to have less health literacy, complicating communication with healthcare professionals (12, 13). Multiple other factors tend to play a role in the adverse health outcomes experienced by Francophone seniors, including sociodemographic factors (i.e., poverty) and cultural factors (i.e., services not well suited for them) (13). For example, compared to Anglophone seniors, they have less access to health-related services at home and in hospitals (14, 15). Thus, when considering the heightened health risks faced by Francophone seniors, it becomes clear that further research is needed to protect this population.

Location of residence is also essential to health disparities among older populations, as people living in rural and remote areas experience a lack of access to health services and preventative programs. Seniors residing in NB have the highest prevalence of chronic health conditions (such as hypertension, arthritis, chronic pain, depression, diabetes, heart disease, and stroke) in Canada (16). Indeed, nearly 67.5% of New Brunswickers have multiple chronic conditions, with 42.5% of seniors living with three or more chronic health conditions and 23% of seniors taking six or more prescribed medications regularly (16). Chronic health conditions are very costly and resource-sensitive due to the need for care and medication; they also greatly impact the quality of life (17, 18). When health services and preventative programs are not tailored to the needs of the aging population a considerable social and economic burden is observed at the systems and community and family levels. In 2020, NB spent about 16% of the province’s gross domestic product on health (GDP, the total value of all goods and services produced within 1 year in NB). This health spending in NB exceeded the Canadian average (13.8%) (19). Older adults who experience loneliness and social isolation are more likely to have poorer health-related quality of life, detrimental effects on mental health and wellbeing, and moderate-to-severe levels of frailty, which increases their risk of institutionalization and mortality (20). Community-dwelling seniors living in rural areas report worse health outcomes, higher morbidity rates (21), and lower quality of life and social functioning (22) than their counterparts residing in metropolitan areas. Seniors living in rural areas in NB have access to fewer service providers, meaning fewer support options for seniors (23). Adequate and appropriate PA can substantially attenuate these consequences of aging. Additionally, PA has a vital preventive role for many chronic diseases (such as cardiovascular disease, diabetes, stroke, and osteoporosis) (24, 25), mobility improvement, fall prevention (26, 27), mental health (28), and wellbeing (29).

PA has been defined in different ways. The most widely used definition of PA was provided by Caspersen et al. (30), who defined PA as “any bodily movement produced by skeletal muscles that results in energy expenditure. This definition framed PA solely as a mechanistic action of the body and only connected it with energy expenditure. However, a broader definition of PA is emerging to emphasize the holistic nature of it. The new, broader definition of PA is proposed as involving “people moving, acting and performing within culturally specific spaces and contexts, and influenced by a unique array of interests, emotions, ideas, instructions and relationships” (31).

The Canadian National Movement Guidelines outline national-level standards of PA. For individuals 65 years and older to be considered physically active, they must meet the following three criteria: (1) performing physical activities (at least 150 min of moderate-to-vigorous PA including muscle-strengthening activities at least two times a week and physical activities that challenge balance); (2) getting adequate sleep (7–8 h per night); and (3) limiting sedentary behaviors (8 h or less including, 3 h or less of recreational screen time with repeated breaks to avoid prolonged sitting) (32). Evidence shows that exercise intervention programs improve low body mass, muscle strength, mobility, energy, and cognition (33), thus optimizing the quality of life and wellbeing (34). There is strong evidence for using PA and exercise as therapeutic approaches in pathological conditions and as preventative approaches for healthier seniors (35).

Most research has focused on the outcomes of the PA program among older adults in rural communities. Planning and implementing a community-specific PA program could be challenging and affected by various factors. Factors that affect implementation of PA programs targeted for a specific population in those communities could be individual, program, or environment-related and are context-specific (36). Insights into those unique challenges are crucial for developing and implementing a successful PA program. It is also essential to understand the barriers and challenges seniors face in rural areas when engaging in PA. Given the need to know the key strategies to promote long-term engagement in PA among community-dwelling seniors in rural areas, the socio-ecological model (SEM) provides a solid theoretical foundation for understanding how various factors at different levels influence human behavior (37). SEM is widely used in public health research to understand the complex interplay of factors that influence human behavior by considering multiple levels of influence—individual, interpersonal, organizational, community, and policy. Indeed, SEM allows for a more comprehensive understanding of social and behavioral issues, which is crucial for designing and implementing effective context-sensitive PA programs. Thus, the aims of this study are twofold: (1) to gain, using the SEM, a better understanding of the needs, barriers, and motivations for engaging Francophone community-dwelling seniors in PA programs in a rural area of NB and (2) to derive recommendations to inform the co-design of a PA program for seniors living in that rural area.

## Materials and methods

### Study design

A qualitative study design using a unique case study through a phenomenological perspective to better understand the perceptions and experiences of francophone rural community dwelling seniors regarding PA. Content analysis of interviews and discussions conducted with the participants was used for this purpose. Qualitative research is recommended when researchers seek to answer questions about the feelings of participants, how they perceive something, and what something is like for them (38). Thus, considering the research question explored in this study, a qualitative approach was the appropriate design to examine the perceptions of community-dwelling seniors related to their needs and motivations for engaging in PA while remaining valid and rigorous (39). Findings from this study are expected to inform the codesign and co-implementation of a PA program for Francophone community-dwelling seniors in a rural area.

The COnsolidated criteria for REporting Qualitative research were used to structure this study andensure its methodological quality (Supplementary File 1)

### Participants

Participants were recruited in a rural area (Memramcook, New Brunswick Canada) through purposive and convenience sampling, utilizing a snowball technique. Collaboration with a civil servant working at the municipality was done to identify the first list of key informants. The snowball technique was used to expand the purposive sample as the data collection was being done until saturation of information was reached. Except for two study participants (program managers working with the municipality), the entire sample lived in the rural area. The participants were approached in person or through the local journal and flyers displayed in areas frequently visited by seniors (i.e., the seniors’ club, the “Ladies’ group,” the Knights of Columbus, the gas station, and the grocery shop). For community dwelling seniors, inclusion criteria was speaking French, living in the community or in the nursing home located in this community, and being available to attend a group discussion in-person. In total, 24 participants were recruited, signed a consent form, and completed a short social-demographic questionnaire. No financial compensation was offered, but a light snack was provided, and transportation was organized when needed (carpooling). Ethical approval was obtained before proceeding with data collection.

### Data collection

We used semi-structured interviews to collect data among managers working with the local municipality, as we were not able to find a convenient time to organize a focus group. All data from community dwelling seniors was collected using focus groups. Data collection (interviews and focus groups) was conducted by the first author (JJ). The interviewer is a female professor (MD, PhD) with strong qualitative research experience with older adults and expertise in conducting interviews. No relationships with the participants were established before the interviews. The data collection was conducted in places that were most convenient for the participants (including an accessibility ramp), as mobility can sometimes become an issue within this population. At the beginning of the discussion, a briefing was given to participants regarding the reasons for doing the research and the importance of the subject. Still, no personal characteristics were shared about the first author or their objectives for research with older adults. Before the interviews, participants had to complete and return signed consent forms and a short sociodemographic questionnaire. However, as the literacy level is lower in rural areas, all written documents were read and explained by the interviewer and verbal consent was obtained before the interview with participants who did not return the signed documents. When needed, the interviewer administered the short sociodemographic questionnaire orally to overcome literacy challenges. In total, three interviews were conducted with individuals with low literacy levels (two males and one female). As the purposive sample was inclusive, it allowed us to hold nine in-person semi-structured interviews (with individuals who do not have access to technology), 3 through Teams (Tech savvy participants) and 1 by telephone (individual with limited mobility capacity). Focus groups were also held with the Ladies’ group (eight female participants, including a person with an early stage of cognitive impairment and a caregiver of a person experiencing cognitive impairment) and with the Knights of Colombus (three male participants). All focus groups were held in person in the village. The interviewer and the participants were the only people present during the interviews, for focus groups and individual interviews. The interviews lasted 47 min on average (minimum 23 min; maximum 136 min).

An interview guide, developed specifically for this study and built on a review of the literature, was used to collect data and gather insightful information to answer research questions. A first draft of the interview guide, written in French, was shared with the co-authors and the municipality representative for content validation, clarity, and intelligibility of the questions and its feasibility (time of administration). A revised version was approved by the team and submitted for ethical approval. However, it was not pilot-tested with community-dwelling seniors. Regarding its content, the interview guide (Supplementary Files 2, 3) included precise instructions for the interviewer to standardize the data collection process, a set of open-ended questions and main subjects to cover. It was designed with four key sections to cover participants’ points of view and to inform the codesign and co-implementation of PA programs in this village. First, an icebreaker question was used to introduce the conversation around PA and participants’ perceptions and knowledge about its benefits and challenges. Secondly, the expectations and needs of community-dwelling seniors and their readiness to engage in PA programs were assessed. Then, the conversation focused on existing and required resources to support regular engagement in physical activities. Finally, suggestions and ideas were discussed regarding the best ways to motivate community-dwelling seniors to engage in regular PA. The same interview guide was used for semi-structured interviews and focus groups. During the interview, the first author guided the conversation toward key subjects, allowing participants to discuss their needs and perspectives freely. No repeat interviews were done with the participants. All interviews were audio recorded but not video recorded. Data collection continued until information saturation was reached.

### Data analysis

A content analysis approach was utilized, as recommended by Miles et al. (40). Content analysis is an inductive qualitative analysis that allows for describing patterns without a-priori theories or models to guide the analysis. The audio-taped focus groups and semi-structured interviews were analyzed manually using an Excel grid developed by two analysts (JJ and ID). A numerical code was created for each participant, and personal information was removed to anonymize data. The analytical approach involved multiple iterations of content analysis, with codes developed through open and axial coding to identify emerging themes. A codebook was developed to ensure data analysis consistency. The two analysts met regularly to discuss the coding process and control for subjective interpretation. A deductive method was then used to examine and categorize codes, included in each merging theme, to the corresponding SEM level: individual, community, and organizational (37). The individual level refers to personal factors including age, gender, knowledge, attitudes, culture, beliefs, behaviors, and preferences. The community level includes social relationships and interactions with individuals, including family, friends, and neighbors. The organizational level refers to institutions and community-based organizations including seniors clubs, municipalities as well as social networks (e.g., neighborhoods, local clubs, social groups). The organizational level includes policies, laws, and structural dimensions of the program (e.g., transport systems, and financial support, as well as PA program components and strategies for recruitment and retention).

Data analyses and syntheses allowed the development of a set of recommendations, including an initial version of the PA program for community-dwelling seniors and strategies to support its implementation in this village. A validation process was organized with the participants and a larger audience. The first author prepared two presentations. A community-based in-person meeting was organized in the village, and all study participants were invited. The first author shared a lay language presentation of the findings and their implications for the design and implementation of a PA program. Participants had the chance to reflect on the structure and content of the proposed program. They also had the opportunity to share their suggestions on strategies to enhance the inclusiveness of the proposed program, as poverty and mobility issues represented the major barriers to access to PA in the village.

The lay audience validated the structure and the content of the proposed program. This meeting lasted 3 h. A second 1-h virtual webinar was organized by the last author (principal investigator of the project) to share findings with key stakeholders, including government and local representatives, community members, and academics. The meeting was intended to gather useful suggestions to tailor the proposed PA program better.

## Results

### Sample description

In total, 24 participants were recruited and interviewed in this study. The mean age of community-dwelling seniors participating in this study was 75.5 years old (min = 65; max = 88). Participants reported a wide range of levels and types of PA and perceptions about being active and engaging in PA programs. The purposive sample was diverse and heterogeneous to capture the perspective of Francophone community-dwelling seniors living in this village. The social-demographic characteristics of the participants are presented in more detail in Table 1.

**Table 1:** Characteristics of study participants.

Participant characteristics	
Mean age, years (SD)	75.5 years (6.34 years)
Sex, *n* (%)	Male = 8 (33%)
	Female = 16 (66%)
Place of birth (Province, Country)	New Brunswick, Canada (100%)
Marital status, *n* (%)	Married = 18 (75%)
	Widow = 3 (12.5%)
	Couple = 2 (8.33%)
	Single = 1 (4.17%)
Level of education, *n* (%)	College = 11 (45.8%)
	University = 6 (25%)
	High School = 5 (20.8%)
	Grade 9 = 2 (8.33%)
Average annual income, *n* (%)	$20–39,999 = 6 (25%)
	$40–59,999 = 10 (41.7%)
	$60–79,999 = 6 (25%)
	$Over 90,000 = 2 (8.33%)
Average weekly physical activity (SD)	338.04 min (297.82 min)
Intensity of physical activity, *n* (%)	Weak = 7 (29.2%)
	Weak to moderate = 5 (20.8%)
	Moderate = 8 (33%)
	Moderate to intense = 2 (8.33%)
	Intense = 2 (8.33%)

### Data synthesis and conceptualization

An inductive thematic analysis of the gathered qualitative data highlighted three emerging themes, categorizing factors that may impact participation in PA programs among community-dwelling seniors living in rural areas. Themes included challenges and barriers, motivators and incentives, and strategies to improve recruitment and retention. The first two themes describe what could potentially limit or motivate participation. The last theme combines participants’ suggestions to make a program attractive, accessible, and adapted to their needs and preferences.

Codes included in the emerging themes were then categorized using a deductive approach to classify them by level, as outlined in the socio-ecological model (SEM). The codes were discussed corresponding to the individual, community, and organizational influences upon PA programs for francophone community dwelling seniors living in a rural community to derive practical recommendations. Major themes and SEM levels are summarized in Table 2.

**Table 2:** Major themes identified in the interviews.

Themes		Examples provided by the participants
Challenges and barriers	Individual level: personal challenges	Physical health issues and co-existing conditions Psychological factors Lack of long-term commitment to complete a program Lack of access to technology Financial challenges
	Community level: social and cultural challenges	Social isolation Poverty Stigma
	Organizational level: community and environmental challenges	Unfavorable transportation and lack of public transport facility Harsh weather conditions and unique geography Inaccessible infrastructure Lack of resources to maintain community infrastructure Lack of shared services and resources
Motivators and incentives	Individual level: demographics	More willingness to participate among women Motivation among couples in their 60s, adults between (40–60 years old), and active individuals People living in apartments or nursing homes are more likely to participate Participants with family and peer support are more likely to participate Francophonic community
	Individual level: health literacy and understanding the benefits of the program	Understanding the physical and psychological benefits of PA encourages participation
	Community level: sense of belonging	Inclusiveness and diversity in the program participants will encourage Tailoring the program as a social event Intergenerational involvement is also suggested as a motivator
	Community level: wide range of physical activities	Individual and group activities of different intensity levels and Inclusion of leisure activities will encourage more participation
Needs and expectations of participants on designing the program	Community level: infrastructure	Safe and accessible location
	Individual level: preferences—contents of the program	Inclusion of different types of physical activities Incorporating educational sessions and other helpful content based on the needs of participants Additional activities like gardening or birdwatching can be part of the program
	Individual level: preferences—structure of the program	Tailored activities according to the needs and capabilities of the participants Rotation of the program in different areas of the community to ensure broad participation Acceptable duration with different intensity levels
	Individual level: preferences—time of program offering	Offering the program at certain times of the day and the year will encourage more participation
	Organizations level: preassessment of the capability of participants	Evaluation of health conditions before enrolling
	Community level: feeling of familiarity	Familiar people in smaller groups Selecting community member as an instructor Incorporating music during exercise Intergenerational participation
	Individual level: preferences—recruitment and retention strategies	Recruitment through both active and passive methods Providing information about program contents and benefits Including information/trial sessions Tailored for different capability levels of participants Making interesting program design Incorporating music, dance, snacks/meals Including social celebration/recognition and outdoor activities Forming small groups Providing transportation to participants (policy level) Offering the program at low-cost or free (policy level) Involving other community organizations (organizations level)

Quotes are included to illustrate key points and were tagged using a participant numerical code (i.e., P1, P2) to maintain anonymity and confidentiality. Quotes were translated from French into English for this publication.

### Challenges and obstacles to access to physical activity programs

#### Community and environmental obstacles

Living in rural areas presents major challenges regarding access to services and programs. Among these, geographical accessibility remains the most difficult to overcome. Participants described the community of Memramcook, New Brunswick, Canada as having an important division because of its large territory, which is 145 km long and subdivided into three rural areas with very low population density. These subdivisions are geographically different (i.e., valley, mountain, and waterfront) and claim different cultural and social belonging. However, all participants reported similar challenges. First, the size of the territory does not favor active transportation (i.e., walking, wheelchairs, or bicycles), and there is an absence of public transportation. Although a free public transit program managed by local volunteers is available, few seniors use it out of pride or fear of stigma. When participants were asked what resources would be needed to participate, it was stated: *“a bus”* (P1). Second, the area’s rough terrain, the lack of sidewalks, and the quality of existing roadways are also of great concern. Moreover, due to meteorological conditions (i.e., snowstorms) and environmental constraints, a large portion of the roads are built from gravel or dirt, which are potential safety hazards for PA. The scarcity of resources impacts winter road maintenance (i.e., snow removal and de-icing), making it hard for seniors to get out in the community. Third, the social competition between the different subdivisions causes a lack of unity in the community (i.e., lack of collaborative projects or sharing services and resources). Challenges related to living in rural areas were also reported by some participants, such as the lack of accessible infrastructures (i.e., the absence of wheelchair ramps and narrow doors). This was reflected in the interview:

*“We have difficulty finding a room that is accessible to all”* (P3).

*“There is only one place where we can do physical activity, the rest of the places are not accessible via wheelchair”* (P4).

In rural areas, lack of funding remains a major obstacle to improving accessibility to PA facilities.

#### Personal challenges

Participants also reported numerous personal challenges, primarily related to physical health (i.e., heart condition, osteoarthritis, pain) and physical limitations (i.e., functional incapacity, disability) that prevented them from participating in PA programs. Seniors experiencing cognitive decline have psychological factors that were also discussed, such as the lack of motivation to participate in physical health programs, the lack of self-confidence due to inadequate practice, the fear of being judged by others, or not having the capacity to accomplish the exercises. In participant’s words:

*“Most of the seniors who participate are in good health, maybe it is those who don’t participate who experience more challenges”* (P1).

Many participants mentioned that most PA programs require long-term commitments (i.e., 3–6 months) which makes the program less attractive to them. Another important challenge mentioned was technology literacy and not being tech-savvy. Some seniors do not know how to use technologies, and others do not have access to electronic devices (i.e., tablets or computers) or high-speed internet, as indicated here:

*“We would possibly lose half of the participants, as many of the seniors don’t have the necessary capabilities with technology”* (P6).

Accessibility to financial resources (affordability) was mentioned as another major obstacle. Many seniors face socioeconomic challenges and cannot afford PA-related expenses (e.g., session fees, sports equipment, and transportation).

#### Social and cultural challenges

Participants discussed social aspects and vulnerabilities as significant obstacles in this rural region. Social isolation and poverty are the most reported challenges. It was noted that, no matter what activity is proposed, the same people always participate in all these activities, which can be somewhat intimidating for those who are novices in PA. It was stated that:

*“It is always the same people participating in activities, which does not leave much room for diversity”* (P10).

Despite these challenges, participants reported that seniors are typically more involved than younger generations. Their community is very generous and helpful to others and is engaged in welfare activities that they recognize as having socially redeeming values (e.g., volunteering, donation, home visits, and carpooling), as stated:

*“In this community, the people are very generous and eager to help others”* (P9).

### Motivators and incentives to participate in physical activity programs

#### Demographics

Motivated seniors who feel capable of participating in PA programs and have support from their peers and family are most likely to participate. P5 mentioned that those who had values of PA during their childhood and perceived being active as an essential aspect of their life are more likely to participate in PA programs. Women in the community were more likely to participate in organized activities or programs (may represent 70–100% of attendees) than men. Couples in their 60s, adults between 40 and 60 years old, active individuals, and people who live in apartments or nursing homes were more likely to participate in any program, including PA programs. In this community, most individuals identify themselves as Francophones and are proud of their language and culture. However, participants agreed that programs delivered in official languages or offering simultaneous translation should be encouraged to overcome language barriers for newcomers and the Anglophone community and facilitate program adherence and engagement.

#### Understanding the benefits of the program

Participants mentioned that understanding the program’s benefits was key to encouraging participation. They were more likely to participate in the program, which would benefit their physical and psychological health and give them a sense of accomplishment. It was said that:

*“It is important to bring the seniors to understand the importance of participating in such programs and to belong to such groups, the participation in activities can come from that”* (P9).

#### Sense of belonging

Participants mentioned the need for the PA program to promote inclusivity and diversity to increase participation further. More specifically, marginalized groups, isolated elders, people with special needs, retired adults, adults with cognitive difficulties, and people speaking different languages should be included in the program.

The social aspects of the program were also discussed as a key factor in encouraging participation in a PA program, further highlighting the need for connection and belonging. For older people, the social aspect was emphasized as being essential to make the program attractive. Participants discussed the importance of a program being displayed as pleasant and social, increasing their sense of belonging by fostering mutual support and group motivation between group members. Multiple participants corroborated this:

“S*eniors love social activities… like getting together to meet other people, drink tea, or listening to a conference… you know… it can be less intimidating for them when they have never done such an activity (Physical activity programs) before”* (P6).

#### Preferred physical activities

The participants expressed interest in various physical activities where most are individually based (such as walking, running, weight training golf, stretching, skating, cycling), and some are group-based (e.g., Zumba, line dancing, yoga, bowling, pickleball, mini golf, and softball). They also enjoy other leisure activities unrelated to PA, such as chopping wood, playing darts and pool, hunting, fishing, gardening, watching performances by children in their community, participating in activities with younger generations, playing pucks, playing cards, and watching different sports. It was very important for participants that the activities were diverse, suitable, entertaining, and fun for a population of community-dwelling seniors. Though numerous activities were mentioned, a lack of interest in activities delivered as part of the program could determine whether they are more likely to participate in the program or not:

*“We do not want activities that are too intense or competitive”* (P7).

### Designing programs to meet the needs and expectations of community-dwelling seniors

#### Where: infrastructure

Participants suggested that the program should be offered in a large, accessible, and safe place. Unfortunately, this rural community has limited options to meet these needs, and most infrastructures have physical barriers for people with mobility challenges. For instance, many buildings have stairs, which are simply inaccessible for individuals with physical difficulties. However, participants mentioned the gym facility at the elementary school, the ballroom at the municipality, and the seniors’ clubs as potential spaces that can be used to deliver PA programs for seniors. Among these spaces, only one is not accessible for wheelchairs. It was mentioned that:

*“We have two available places, but both have costs associated with their rent”* (P9).

#### What: content

Participants suggested various activities that should be included in the PA program. Some recommended stretching exercises, balance exercises, weight training or cardio (treadmill or bicycle), gentle aerobics, dancing, walking, and swimming. It was also proposed that equipment such as stationary bikes, treadmills, and machines for weight and strength training could be included in the program. The addition of short educational conferences (10–15 min) was recommended:

*“Videos regarding subjects such as nutrition, diabetes, heart problems, mental health symptoms, and cognitive disorders would be interesting”* (P5).

Suggestions were made to include different helpful contents, such as legal requirements (e.g., how to complete their will), agriculture, or technology. Activities like bird watching or gardening incorporating intergenerational contact (e.g., pairing a child with a senior to do an activity like gardening) were also discussed.

#### How: format structure

Participants suggested that the physical activities should be diverse, and the program should be flexible and adjusted according to the participant’s needs. The program should be accommodating if someone cannot participate in one session because of health reasons, social responsibilities, or even transportation difficulties. There should be equity and harmony between the three subdivisions of the region of the rural community. The program sessions could rotate between the three areas with access to transportation for participants to travel to the different areas, or sessions could be offered in all regions simultaneously:

*“The program must be both flexible and open to changes”* (P2).

Regarding the structure of the program session, participants mentioned that stretching and balance exercises could start the sessions, followed by physical activities, and then end with complementary activities such as short educational conferences or social activities. A few suggested adding cognitive training activities or counseling for psychological wellbeing and stress management. The program duration should be between 4 and 8 weeks, with 1 or 2 sessions a week ranging from 45 to 60 min each. Certain participants mentioned that a 12-week program would be acceptable if subdivided into different levels. A program offered in progressive stages or a circuit-type program that includes all capabilities was also recommended.

#### Time of offer

The participants brought up an interesting aspect regarding the optimal timing of the program delivery. They suggested that the program should take place from November to May. Participants are more likely to participate during this time because they have more availability and fewer implications in the winter (e.g., weather) than in the summer. The sessions should take place in the late morning or early afternoon to accommodate the needs of most elders. Many elders mentioned that they often have activities after supper, limiting their ability to add more programs.

#### Evaluation of capabilities

Participants also mentioned that it would be beneficial to have an initial consultation to evaluate physical and health capabilities before the start of the program will ensure their ability to participate. This knowledge would give the instructors and the participants a sense of security and confidence that they can do the exercises included in the program, as mentioned by the participants:

*“We need to look at our strengths, our weaknesses, what we already have, what we don’t and how do we get them”* (P4).

#### Feeling of familiarity

Participants suggested the program should be offered in small groups by a local community resident. This would provide a feeling of familiarity, making them more comfortable with the program. Other elements to increase their familiarity include incorporating music from their generation or having intergenerational contact with them. Participants expressed their desire to have fun in a program, thus allowing further participation, as stated:

*“Seniors really enjoy dance classes, yoga, activities with music”* (P6).

### Recruitment and retention strategies

Various recruitment strategies were suggested, including passive methods (i.e., social media, the town’s newspaper, posters in popular areas such as arenas, churches, in the church bulletin, flyers, and on the radio). Recommended active methods included calling individuals directly, word-of-mouth (from friends and family), personal letters, or communicating with and through existing groups or community leaders. It was also suggested to have younger people do the recruiting to encourage participation. Efforts should also be made to identify isolated and low-income seniors and recruit them to participate, which could be done by community groups like the wellness committee. A sense of worry was expressed regarding other seniors who live in isolated communities. It was mentioned:

*“If we start a program, we need to find a way to attract the people that stay at home, but this may be difficult because often they don’t want to be bothered”* (P9).

For effective recruitment, the content of the programs should be clearly explained so that they know what to expect and can make an informed decision on whether they would like to participate. Also, there should be information on program content or trial sessions to allow participants to try it before committing to the program. Developing short information videos and infographics on the benefits of PA could effectively recruit people into the program. The content should be adapted to the different groups of people based on their PA level (e.g., active vs. not very active or not active at all), as stated:

*“Many seniors have cognitive issues in the region, we could find a way to include them and to adapt the programs”* (P6).

Participants suggested specific strategies and incentives to encourage their retention in the program. Transportation and carpooling should be developed to facilitate accessibility and program engagement. The importance of maintaining interest from one session to the next session was also highlighted. The program needs to be positive and include social dynamics among the groups, professionals, and instructors. Some mentioned that having enjoyable competitions would encourage participants to stay in the program, but others thought competition and rivalry should be avoided. Most participants recommended including music and dancing to emphasize a pleasant environment for retaining them in the program, and some also suggested including a snack or a healthy meal during the sessions. Other recommended motivators were celebrations, prizes, personal recognition, and encouragement to foster a positive and supportive approach throughout the program. If participants feel encouraged and see their progression throughout the program, they will be more motivated to stay throughout the program. It could be more inspiring if they were paired with others to encourage each other to remain in the program and attend every session together, highlighting their desire for accountability and social support. They also mentioned having a reminder system to prompt participants of upcoming sessions to encourage attendance.

Some participants suggested the program should be free with the option to donate or should have it at a low cost (e.g., $5–10). It was emphasized that no one should be refused into the program due to lack of affordability. This would promote inclusivity and respect for those who are less fortunate and address financial barriers to participation. It was stated that:

*“Some people do not have much money, no pension plans”* (P5).

Collaboration and harmonization with other community activities, the wellness committee, and the seniors’ residences would also encourage participants to stay in the program. Finally, some participants suggested that organizing small outings for one program session (e.g., *“a group hike”*) (P6) could also be compelling to include and promote program retention.

The two validation meetings were instrumental in favoring ownership of the proposed program among key stakeholders and facilitating implementation. Participants validated the structure and contents of the proposed program. In those meetings, two aspects were discussed: the location where the program would be delivered and how Francophone culture could be linked to the program. Thus, the leading suggestions consisted of making the program available at no charge. Donations will still be accepted to support the program, physical apparel kits (i.e., tee shirts, shorts, and sneakers) will be available for free, and carpooling will be offered by volunteers participating in the program.

## Discussion

This study aimed to gather an in-depth understanding of factors that may support or prevent community-dwelling seniors living in a rural region from engaging in PA programs and to inform the design, implementation, and evaluation of a tailored PA program. Results are discussed using the SEM levels to derive practical and context-sensitive recommendations at each of the considered levels: individual, community and political.

### Individual level

#### Personal challenges

Seniors living in rural areas often face a variety of significant challenges that can impact their ability and capacity to participate to PA programs. The results of this study confirmed the current literature that many rural seniors experience financial strain (41, 42). However, most of them are often reluctant to use available resources and support programs such as subsidized transportation program or ask for help from friends and family because they feel embarrassed or have a negative perception from others, particularly in a small town where everyone knows each other. Literature is scarce in what are the most effective strategies to put in place to alleviate the stigma associated with poverty and access to supportive resources for seniors experiencing poverty in rural areas. Our findings shed light on several innovative strategies to put in place. For example, community-based carpooling was proposed, as an alternative strategy to governmental subsidized transportation, as it builds upon community solidarity and is orchestrated around the PA program schedule. While carpooling can contribute to improve access to PA programs, it is also considered as an impactful strategy to encourage seniors’ participation and retention in PA programs. Indeed, the drivers can play a key role in creating a social dynamic favoring compliance with the PA program.

Seniors reluctance to commit to a long-term engagement in a program had been identified as a major barrier to compliance to PA programs (41). In this study, several factors were mentioned, including the fear that certain events, such as health problems, personal engagements (e.g., family events), or medical appointments, would interfere with their ability to participate in the program. The findings revealed that personal challenges such as physical conditions (e.g., chronic health problems), discomfort, lack of motivation, and long-term commitments could act as barriers to PA program participation, which is consistent with previous studies (43, 44). These key barriers need to be considered when designing PA programs, such as including motivational interviewing approaches in the initial assessment. However, few studies have explored techniques and benefits of tailored motivational interviewing among senior adults, and specifically among those living in rural areas. Further, a recent systematic review of qualitative studies in this realm reported individuals’ functional capacity (e.g., strength) and perceived risk of injury and pain from exercise (e.g., due to falls) as key factors affecting older adults’ capability and motivation to engage in PA programs (45). Another study by Dobson et al. (46) has indicated that older adults sometimes have concerns that physical exercise can aggravate their existing health conditions (such as joint pain and arthritis, dyspnea or tachycardia). Therefore, highlighting the need to improve health literacy, in general and in specific to the individual’s health condition(s), including education about the benefits and safety of physical exercising. PA programs may include short sessions where participants can discuss their beliefs and fears related to the potential negative impacts of exercising on their health and dissipate negative beliefs about PA. This aligns with findings from this study, as health literacy was identified as a driver, and participants demonstrated a strong knowledge about the importance of PA and active living for their health and wellbeing.

Findings from this study also show that the seniors reported challenges using technology and demonstrated a low level of eLiteracy and reluctance to use electronic solutions. Nonetheless, the use of technology in PA programs (e.g., smartphone apps, online exercise videos) has increased in recent years. Recent literature has demonstrated that digital technologies are acceptable to older people, and are associated with good adherence to PA, suggesting this approach can promote PA among older people (47). Indeed, participants in our study mentioned the usability of digital wearables, such as smartwatches, for tracking daily steps and monitoring heartbeat and breath frequency, as well as to reassure them. They were open to use wearables and share their data with the facilitator of the PA program.

#### Importance of health and physical capacities

An interesting aspect that emerged in this study is that older adults in the Memramcook region are apprehensive about their health. A recent systematic review of qualitative studies identified this concept, highlighting that some key factors, such as certain sensations (e.g., pain) and perceived risk of injury from PA (e.g., falls) could negatively impact older adults’ motivation to engage in PA programs (45). This study also reported that if the participants perceive health gains and experience positive emotions (e.g., enjoyment), they will be motivated to engage in PA programs. Participants of the current study mentioned similar concepts. In addition, they expressed that they could get hurt while doing the program and lose their physical capacities and autonomy. Therefore, including an initial clinical assessment of their health and physical capacities and clinical supervision before and during the program would alleviate this fear of health decline and make participants feel safe. Incorporating this initial clinical assessment and supervision reinforces the importance of considering a PA program for seniors not only from an entertainment angle but also a clinical one, further supporting the program’s benefits.

### Community level

#### Geographic aspects

The geographic and social divisions in the community in Memramcook region are significant because of its vast territory and small population. Participation can be more difficult for older people because of the large territory and the lack of transportation. Several articles have reported that barriers to exercise programs for community-dwelling older people in rural communities include a lack of access to facilities and transportation (36, 48, 49). Transportation barriers can greatly affect participation among this population, with many older adults not having driver’s licenses or their own vehicles (41, 50).

Another finding that emerged from this study is the importance of feeling of familiarity. A study by Dare et al. (41) also found that participants feel more comfortable in a program when other local community members are included. Therefore, if the program is outside their area, they would be less likely to participate. These results demonstrate the need to explain to seniors from rural areas that participating in programs outside their area can be beneficial for their health and is not a way for them to disconnect from their areas. Moreover, community-based PA programs are aimed to encourage, enable, and engage the older population to participate regularly in health-promoting activities. In addition, these programs could stimulate social engagement, a factor known to improve health outcomes in this age group (51, 52).

#### Cultural aspects

One interesting new aspect that emerged in this study and has not been found in other studies is the importance of incorporating components relating to the culture of the Memramcook region to motivate participants. For example, combining music and dancing into the PA program would encourage participation since they are central aspects of the Acadian culture present in Memramcook. Research also has demonstrated that music can motivate PA by making it more joyous, less arduous, and more energetic and efficient (53). These are specific preferences of this community and could be an important cultural motivator to participate in the program.

### Organizational level

#### Designing and implementing the PA program

Like previous research, this study confirms that tailoring PA programs to the needs and capacities of seniors is a crucial strategy. Social aspects need to be considered when designing a PA program. Study analyses revealed that the program should be structured around social interactions while recognizing personal accomplishments and progress rather than competition and performance.

Consistent with previous research, the socialization opportunities were important motivators for this population’s participation and program retention (41). Similarly, this study participants mentioned that adherence to PA programs is most likely to be achieved by providing opportunities and embedding time within the program for participants to interact with one another. The inclusion of interactive programs such as intergenerational activities can encourage active aging and enhance physical function (54). Studies revealed that the availability of social interaction and social support between participants of a given program also enhances feelings of self-efficacy for the seniors and acts as a motivator toward participating in a program (42, 45). Like these findings, the sense of belonging and the inclusivity of the program are important values to implement among seniors and can affect their acceptance to participate in PA programs outside of their area (41). Thus, social aspects of fostering a sense of belonging have been demonstrated by various studies as an important aspect to include in programs, especially for older people in rural areas (41, 42).

Another interesting new aspect that emerged from this study is that participants were more interested in their accomplishments rather than competition and winning. For this population, the competition was associated with increased stress. The study participants expressed that focusing on individual performance rather than comparing themselves to others would increase their motivation and commitment to the program. One review article reported that identifying as an “exerciser” could motivate seniors to participate in a PA program (45). These results suggest that the program needs to be focused on the accomplishments of individuals, with the support of others, rather than focusing on competition among participants.

#### Communication channels and recruitment strategies

To advertise the program, specific communication channels were reported as being more popular for seniors in the Memramcook area. For this population, the best ways to promote the program would be through existing local platforms, such as newspapers, posters, or flyers in popular areas or through existing committees. Recruitment could also be done through social media, but participants explained that this method would be less effective due to limited use and experience with technology.

For the recruiting and promotion of the program, the information must be accessible to older adults and tailored based on their needs and preferences. Hardy and Grogan (53) found that older people often express a lack of appreciation for their needs when such information is not tailored to their needs. Therefore, it is important that the information shared regarding the promotion of the program is accessible and adapted to the comprehension and needs of older adults. This will help them to make an informed decision about their participation in the program. Another important finding that should be included in recruitment strategies is an explanation of program benefits. Consistent with Dare et al. (41), participants who believed participating in the program would benefit their mental health were more motivated to participate. Using the popular communication channels among this population and integrating accessible and key information (e.g., health benefits) are important aspects to consider while recruiting participants from this community. Engaging older adults as proactive and empowered participants is also important while promoting the PA programs. Hence, communication and media should ensure that the exercise program will include physical activities within the bounds of their individual physical capacity and preferences.

#### Importance of collaboration and proximity within rural areas

A collaborative approach would be beneficial when building a PA program because of the physical challenges related to rural areas regarding infrastructure and available resources. These structural barriers to participation, regarding availability and location, limit the opportunity of having local PA programs in rural areas (41). Therefore, it would be important to collaborate with local organizations, such as municipalities, schools, and churches, that currently have resources available to implement a physical health program in a safe and accessible place. Hardy and Grogan (53) also highlighted the need for accessible exercise facilities for older adults who may experience functional limitations and use walkers or wheelchairs. Consistent with their study, this study’s findings illustrate the importance of having accessible facilities for people with impaired mobility to encourage inclusion and participation. This collaboration will help with access to infrastructure and provide the necessary resources to support the implementation of PA programs.

Another important aspect of ensuring the success of PA programs for seniors is offering them near communities and outside health settings. Consistent with previous research, greater proximity to recreational facilities offering the program and transport access encourages participation among older adults in rural and urban areas (50). Researchers have also found that seniors prefer local programs that are convenient and close to home (41).

#### Structure of the PA program

Another interesting aspect that emerged from this study is the variety of activities that the participants suggested for physical activities, including cardio, strength, and balance exercises. Other complementary activities were also recommended based on their interests to improve the program recruitment further. A review of community-based exercise programs targeting seniors living in rural areas included the incorporation of low-to-moderate intensity exercise (e.g., balance, aquatics, yoga) (55). The suggested list of exercises by the study participants included all these activities. Maintenance and retention are two key aspects that need to be well prepared and thought out as participants may be reluctant to engage for more extended periods (i.e., over 6 weeks commitment).

#### Retention strategies

Participants recommended including small discussions or mini conferences related to taxes, nutrition, or other relevant topics for rural seniors to encourage program retention. Including more diverse activities within the program would be a helpful strategy to keep participants engaged and create a more global approach to their health and needs rather than focusing solely on program tasks and performance. As mentioned earlier, creating social connectedness and enjoyment is essential to consider for encouraging program retention among seniors (41, 42). Dare et al. (41) also found that interest in the activities motivated participants to join the program, but the sense of belonging and connectedness maintained attendance over time. Also, incorporating personal recognition and celebrating progress and accomplishments would be valued among participants and encourage retention. Recognizing the progress and appreciating the efforts would contribute to their intrinsic motivation and feeling of self-efficacy, which is also a significant predictor of continued maintenance of PA for elders (53). Therefore, creating a program that integrates these aspects and is tailored to this population’s needs could encourage participants to complete the program.

#### Inclusivity and adaptivity of the program

Finally, inclusivity and non-judgmental approaches are central values among this population, and their desire to adopt the program is based on individual needs. Participants argued that the program needs to be inclusive and adaptable for different capacities (e.g., for those with reduced mobility). Therefore, it should also be adjusted for people who face financial difficulties by offering free-of-cost programs or pay-as-you-go programs suggested by another study (41) where participants pay at each session if they attend compared to a fixed-cost for the whole program.

The program should be accommodating for all and flexible in considering the needs of its participants. Like previous research, seniors require a flexible program to maintain their appointments or health-related problems and avoid feelings of restriction (41). If seniors perceive the program as too restrictive and less accommodating, they will be less likely to commit, fearing that something will “come up,” that could make it more difficult for them to engage fully.

This study has some strengths and limitations. In terms of strengths, this study provides valuable insights into seniors’ specific needs and challenges regarding physical activities and living in rural areas. The current understanding of the factors influencing the acceptability, effectiveness, and implementation of community-based exercise programs for older adults living in rural areas is limited. The study findings can inform the design and implementation of an effective PA program, considering the community’s unique characteristics, such as demographics, geographic location, and socioeconomic and cultural factors. This study will help identify the barriers that prevent seniors from participating in PA programs in that rural community and the facilitators that can promote their engagement. This information is crucial for developing targeted interventions and strategies to overcome barriers and enhance participation.

As for limitations, this study has been conducted in a specific rural community, which may limit the generalizability of the findings to other rural areas, as the characteristics and needs of seniors in different communities can vary significantly. This study was done before the implementation of PA programs, which means that the findings may not capture the long-term impact or sustainability of the programs. Follow-up studies are necessary to assess the effectiveness and outcomes of the implemented programs. This study gathered inputs from community members who are potential participants; however, inputs from other stakeholders, including community leaders and program providers, would ensure that multiple perspectives have been considered. This will lead to a more comprehensive understanding of the challenges and opportunities for implementing the program.

Despite these limitations, this study will play a crucial role in informing the development and implementation of PA programs for seniors in a rural area of NB. It will provide valuable insights into the specific needs and challenges of the target population, helping to tailor interventions and maximize the potential for successful program implementation.

## Conclusion

In summary, the results of this study highlight challenges faced by community-dwelling older people living in rural areas and their motivators to join and maintain participation in a PA program. It also emphasizes the importance of exploring their needs and expectations regarding PA programs to tailor a program that is adapted and accessible to their capacities and needs. Involving older adults in the process of the development of the program and exploring their opinions, preferences, interests, and challenges they may face will help create a program that they will enjoy and benefit from globally. The findings of this study may be particular to this sample; however, the factors identified in this study could be relevant to implementing PA programs in similar populations in rural areas because of the specific sociocultural context.

## Data Availability

The datasets presented in this article are not readily available because as the information provided in the dataset allows to identify the participants of this study, in order to keep confidentiality, we cannot share data. Requests to access the datasets should be directed to JJ, jalila.jbilou@umoncton.ca.
