# Supplementary material for: Promoting physical activity among community-dwelling seniors living in a Francophone rural area in New Brunswick: a pre-implementation qualitative study
Source: Front Public Health. 2025 Apr 9;13:1498397. doi: 10.3389/fpubh.2025.1498397 (PMC12014462; doi:10.3389/fpubh.2025.1498397)
Supplement: Supplementary file 1 [file Data_Sheet_1.zip › Supplementary File 3_SociodemographicSurvey.pdf]

SOCIODEMOGRAPHIC QUESTIONNAIRE

1- What is your sex?

 Female Male Prefer not to share

What is your gender identity? ____

2- In what year are you born?

3- Where were you born? (Province, Country) NB

4- What is your matrimonial status?

 Single (never married)
 Married
 Couple, but not married
 Divorced
 Widow
 Other, specify ……………………………….

5- What is the highest level of schooling you have completed?

 Never attended school
 Primary school / Elementary school (Grade 6)
 Middle school / Intermediate school (Grade 9)
 High school (Grade 12)
 College
 University
 Other, specify: ______________________________

6- What is your current employment status?

 Retired, please indicate since when: ____________________________
 Still working, please indicate your current job and the number of hours worked per week:
____

7- Thinking of the last three years, what is your average annual revenue:

 Less than $19 999
 $20 000 - $39 999
 $40 000 - $59 999
 $60 000 - $79 999
 $80 000 - $99 999
 More than $100 000

8- Thinking of the last four weeks, how would you evaluate your current physical activity levels:

In total minutes per 7 days:
Please indicate at what intensity:
 Low intensity: Aerobic activities, such as mowing the lawn, taking a dance class, or biking to the store, or strengthening activities, such as yoga or weights and dumbbells,
 Moderate intensity: Moderate-intensity aerobic activity makes you breathe harder and increases your heart rate. You should be able to talk, but not sing. Ex. brisk walking, cycling
 High intensity: High-intensity aerobic activity greatly accelerates your heart rate, and you can only say a few words before you have to catch your breath. Ex. jogging and cross-country skiing.

©Jbilou et coll. 2025
