# Supplementary material for: Promoting physical activity among community-dwelling seniors living in a Francophone rural area in New Brunswick: a pre-implementation qualitative study
Source: Front Public Health. 2025 Apr 9;13:1498397. doi: 10.3389/fpubh.2025.1498397 (PMC12014462; doi:10.3389/fpubh.2025.1498397)
Supplement: Supplementary file 1 [file Data_Sheet_1.zip › Supplementary File 2_InterviewGuideFr.pdf]

Le rôle d'un programme communautaire d'activité physique auprès des francophones minoritaires du Nouveau Brunswick : Pouvons-nous adapter le cerveau à l'aide d'activité physique?

GUIDE DE DISCUSSION

PRÉ-IMPLANTATION

Préambule

Nous vous remercions d'être présent (e) avec nous aujourd'hui et de nous offrir un peu de votre temps.

Le but global de ce projet est de contribuer à l'amélioration de la santé et le bien-être des aînés vivant à Memramcook, et ce, en établissant une meilleure compréhension du rôle d'un programme communautaire d'activité physique sur la santé physique et cognitive des ainés francophones au Nouveau-Brunswick.

Cette discussion a pour but de mieux comprendre les attentes et les besoins des personnes aînées en matière de programme d'activité physique.

Il n'y a pas de bonne ou de mauvaise réponse. Seule votre opinion nous tient à cœur.

La rencontre sera enregistrée sur support audio, ainsi nous serons en mesure de retenir toutes les informations que vous nous partagerez. Soyez assurés que nous ne dévoilerons pas votre nom dans aucun document, ni aucune présentation découlant de ce projet. Les propos recueillis seront traités en toute confidentialité et seule l'équipe de recherche aura accès à cette information.

Passer en revue les formulaires de consentement et vérifier si les participants ont toutes et tous signé un consentement.

Passer les questionnaires sur les données sociodémographiques des participants, puis les récupérer et vérifier s'ils sont tous complétés.

Lancer l'enregistreuse et commencer la discussion de groupe.

© Jbilou et coll 2025

Le rôle d'un programme communautaire d'activité physique auprès des francophones minoritaires du Nouveau Brunswick :
Pouvons-nous adapter le cerveau à l'aide d'activité physique?

<table>
<tr><th>Sujets et questions ouvertes</th><th>Relances et sous-questions</th></tr>
<tr><td colspan="2"><u>Brise-glace</u>
L'activité physique contribue à améliorer la santé physique et la santé cognitive des personnes aînées qui sont des facteurs importants pour leur mieux-être global.</td></tr>
<tr><td>1. Comment décririez-vous la participation des personnes aînées de Memramcook à des activités physiques?</td><td>Demander des précisions :
✓ Niveau de participation
✓ Motivations (pourquoi est-ce que ces personnes participent)
✓ Description générale des participants (Âge et sexe)</td></tr>
<tr><td colspan="2"><u>Sujet 1. Les besoins et les attentes</u>
Les besoins et les attentes des personnes aînées sont nombreux et complexes. Parfois, nous pensons bien connaitre leurs besoins, sans nécessairement les consulter pour concevoir et mettre en place des programmes d'activité physique.</td></tr>
<tr><td>2. Selon vous, quels sont les besoins, en matière d'activité physique, des personnes aînées qui vivent à Memramcook?</td><td>Demander des précisions :
✓ État de santé
✓ Fragilité
✓ Santé mentale et démence
✓ Handicap et invalidité
✓ Âge et sexe
✓ Coût
✓ Transport
✓ Technologie</td></tr>
<tr><td>3. Selon vous, quelles sont les attentes des personnes aînées en lien avec leur participation à un programme d'activité physique à Memramcook?</td><td>Demander des précisions :
✓ Accessibilité
✓ Adéquation
✓ Faisabilité
✓ Coût</td></tr>
<tr><td colspan="2"><u>Sujet 2. Les ressources nécessaires</u>
Afin de répondre aux besoins et attentes des personnes aînées qui vivent à Memramcook, il est important de bien connaitre les ressources nécessaires pour les soutenir à participer à un programme d'activité physique.</td></tr>
<tr><td>4. Quelles ressources seraient nécessaires pour soutenir les personnes aînées qui vivent à Memramcook à participer à un programme d'activité physique?</td><td>Demander des précisions : SOUTIEN
✓ Transport, ressources humaines, logistique
✓ Infrastructure, matériel, budget, techno.</td></tr>
</table>

Quelles ressources seraient nécessaires pour implanter un programme et pour le pérenniser?	

**Sujets et questions ouvertes**	**Relances et sous-questions**
**Sujet 3. Les idées et les solutions**<br>*Les personnes aînées connaissent très bien leurs besoins et leurs attentes, ainsi que les solutions à mettre en place pour soutenir leur participation à un programme d'activité physique.*	
5. Quels moyens devraient être mis en place pour que la plupart des personnes aînées qui vivent à Memramcook participent à un programme d'activité physique?	Demander des précisions : RECRUTEMENT<br>✓ Stratégies de recrutement<br>✓ Marketing social<br>✓ Réseau de contact<br>✓ Médias sociaux?
6. D'après vous, que faudrait-il faire pour motiver les personnes aînées qui vivent à Memramcook à continuer à participer à un programme d'activité physique?	Demander des précisions : RÉTENTION<br>✓ Stratégies de motivation et de rétention<br>✓ Incitatifs<br>✓ Format, durée, organisation<br>✓ Contenu, activités
**Clôture**<br>7. Avant de terminer la séance, y a-t-il des questions ou des sujets que nous n'avons pas abordés et dont vous aimeriez nous parler?	

© Jbilou et coll 2025
